# Plant-Derived Secondary Metabolites Tetrahydropalmatine and Rutaecarpine Alleviate Paclitaxel-Induced Neuropathic Pain via TRPV1 and TRPM8 Modulation

**DOI:** 10.3390/metabo16010046

**Published:** 2026-01-04

**Authors:** Keun-Tae Park, Hyesang Yun, Juyeol Kang, Jae-Chul Lee, Woojin Kim

**Affiliations:** 1 Department of Physiology, College of Korean Medicine, Kyung Hee University, Seoul 02453, Republic of Korea; cerex@naver.com (K.-T.P.); yhsaye@hanmail.net (H.Y.); kmdju2@gmail.com (J.K.); crewera@gmail.com (J.-C.L.); 2 Korean Medicine-Based Drug Repositioning Cancer Research Center, College of Korean Medicine, Kyung Hee University, Seoul 02453, Republic of Korea

**Keywords:** paclitaxel-induced neuropathic pain, *Corydalis yanhusuo*, *Evodia rutaecarpa*, tetrahydropalmatine, rutaecarpine, TRPV1 modulation

## Abstract

Background: Chemotherapy-induced peripheral neuropathy (CIPN) is a major dose-limiting adverse effect of paclitaxel and is characterized by cold and mechanical allodynia. Effective therapeutic strategies for CIPN remain limited. This study evaluated the analgesic potential of *Corydalis yanhusuo* (CY) and *Evodia rutaecarpa* (ER), as well as their major alkaloids tetrahydropalmatine (THP) and rutaecarpine, in a mouse model of paclitaxel-induced neuropathic pain. Methods: Neuropathic pain was induced by paclitaxel administration (2 mg/kg, i.p., four injections). CY and ER extracts were orally administered at doses of 100 or 300 mg/kg, either alone or in combination, and cold and mechanical allodynia were assessed from days 0 to 8. The analgesic effects of THP and rutaecarpine were also examined. Gene and protein expression analyses were performed to evaluate the involvement of TRPV1 and TRPM8 signaling pathways, and high-performance liquid chromatography (HPLC) was used to confirm the presence of THP in CY and rutaecarpine in ER. Results: Paclitaxel reliably induced robust cold and mechanical hypersensitivity. Oral administration of CY or ER significantly alleviated allodynia in a dose-dependent manner, with greater efficacy at 300 mg/kg. Combined CY–ER treatment produced stronger anti-allodynic effects than either extract alone. THP and rutaecarpine also exhibited dose-dependent analgesic effects, and their co-administration yielded the most pronounced inhibition of paclitaxel-evoked hypersensitivity. Molecular analyses confirmed the involvement of TRPV1- and TRPM8-related pathways in these analgesic effects. Collectively, these findings indicate that CY, ER, and their representative alkaloids effectively attenuate paclitaxel-induced neuropathic pain and highlight CY–ER-based natural products as promising candidates for managing CIPN through modulation of TRPV1/TRPM8 signaling.

## 1. Introduction

Neuropathic pain arises from damage to the central or peripheral nervous system caused by diseases such as stroke, diabetes, or multiple sclerosis, as well as toxic insults like chemotherapy or physical trauma [1,2]. Effective treatment aims to prevent disease onset and modulate the mechanisms underlying neuropathic pain [3]. However, existing therapies often provide insufficient analgesia due to dose limitations, reduced efficacy, and adverse effects, highlighting the need for ongoing research into novel antinociceptive strategies [4]. Our previous studies have explored the antinociceptive properties of morphine, bee venom, and various medicinal herbs [5]. Herbal medicines offer advantages for pain management because they are generally safer and associated with fewer side effects compared to synthetic drugs [6,7].

Paclitaxel, a chemotherapeutic agent derived from Taxus brevifolia, is widely used to treat ovarian, lung, and breast cancers [8,9]. Despite its high efficacy, its clinical application is often restricted by dose-limiting peripheral neuropathic pain [10]. Patients typically develop numbness of the extremities, cold allodynia, and mechanical allodynia within 24 h of the first administration [11], leading to treatment discontinuation and reduced quality of life [12]. Therefore, developing therapeutic options that effectively alleviate chemotherapy-induced neuropathic pain while minimizing side effects remains a critical clinical need [13].

*Corydalis yanhusuo* W. T. Wang (yuanhu) (*C. yanhusuo*, CY), a member of the *Papaveraceae* family, has long been used in traditional Chinese medicine (TCM) and is a key ingredient in classic prescriptions such as Jin Ling Zi San (JLZS) and Yuanhu-Zhitong (YHZT). According to the Chinese Pharmacopoeia [14], its dried tuber (*Corydalis Rhizoma*) is the pharmacologically active part, traditionally used for pain relief and improving blood circulation. Its extract contains various compounds, with alkaloids recognized as the major bioactive components [15]. Modern studies have shown that CY alkaloids exert multiple pharmacological activities, including anti-addiction [16], protection against ischemia/reperfusion injury Ling, 2006 [17], anti-cancer [18], and antidiabetic activity [19]. Among these, tetrahydropalmatine (THP) is a key tetrahydroprotoberberine isoquinoline alkaloid naturally found in *Corydalis* and Stephania species [20,21]. THP exhibits antioxidant and anti-apoptotic properties via inhibition of TLR4/NF-κB-mediated macrophage activation [22], and modulates PI3K/AKT signaling to suppress inflammation in ischemic and skin flap models [23] and it improved skin flap survival via the same pathway [24]. Furthermore, THP has shown significant analgesic and neuroprotective effects in neuropathic pain models [25,26], though its mechanisms in neurotrauma remain to be fully elucidated.

The dried ripe fruit of *Evodia rutaecarpa* (Juss.) Benth. (Rutaceae) (ER), a member of the Rutaceae family, has been widely used in traditional Chinese medicine (TCM) to dispel cold, soothe the liver, and relieve pain [27]. It exhibits diverse pharmacological properties, including antidepressant, digestive, analgesic, sedative, antibacterial, and antioxidant activities. Alkaloids are identified as its major active components [28], with earlier research highlighting indole-type alkaloids [29,30]. In TCM, therapeutic effects are attributed to compounds absorbed into the bloodstream rather than the total raw content [31]. Among these alkaloids, rutaecarpine has gained attention for its pharmacological potential. Animal studies showed that rutaecarpine (25 µg/g) significantly reduced mortality in ADP-induced pulmonary thromboembolism, demonstrating potent antiplatelet activity [32]. In human platelet-rich plasma, rutaecarpine (40–200 µM) inhibited aggregation induced by collagen, ADP, and arachidonic acid [33]. This effect is associated with reduced [^3^H]-inositol monophosphate production, suggesting suppression of platelet activation through phospholipase C (PLC) inhibition [34]. Despite its broad pharmacological profile, the analgesic effects of rutaecarpine in chemotherapy-induced neuropathic pain remain unexplored.

In this study, we aimed to investigate the analgesic potential of CY and its major active alkaloid THP, as well as ER and its bioactive compound rutaecarpine, against paclitaxel-induced neuropathic pain. We focused on their potential to modulate the TRPV1 and TRPM8 signaling pathway, which plays a key role in pain transduction. The timing of administration was adjusted to assess both preventive and therapeutic effects on the development and maintenance of neuropathic pain. This study is expected to provide a foundation for developing natural product-based analgesics targeting TRPV1.

## 2. Materials and Methods

### 2.1. Screening of Active Compounds and ADMET Prediction

To investigate the therapeutic mechanisms of CY and ER on Chemotherapy-Induced Peripheral Neuropathy (CIPN), their representative bioactive compounds were selected based on the standards of the Pharmacopoeia. Specifically, l-Tetrahydropalmatine (PubChem CID: 72301) and Rutaecarpine (PubChem CID: 65752), which are the quantitative marker compounds of CY and ER, respectively, were chosen for this study. To ensure drug-likeness, the compounds were further verified to meet the general screening criteria of Oral Bioavailability (OB) ≥ 30% and Drug-Likeness (DL) ≥ 0.18 using the TCMSP database [35]. The absorption, distribution, metabolism, excretion, and toxicity (ADMET) properties of THP and rutaecarpine were predicted using the pkCSM web server (http://biosig.unimelb.edu.au/pkcsm/ (accessed on 22 November 2025)) [36]. Key parameters were evaluated to assess their suitability as CNS-active therapeutic agents. Both compounds demonstrated central nervous system permeability (logPS > −2.0) and blood-brain barrier penetration capabilities (logBB > −1.0).

### 2.2. Identification of Potential Targets for ER and CY and Construction of the PPI Network

To ensure a comprehensive understanding of the therapeutic mechanisms, we performed a dual-level network analysis. First, we identified the targets of the whole herbs (ER and CY). Second, we specifically analyzed the targets of their representative active compounds, rutaecarpine and THP, to validate their key roles in treating CIPN. Potential therapeutic targets for CIPN were collected from the GeneCards database (https://www.genecards.org/) with a relevance score threshold to ensure data quality [37]. To identify the potential therapeutic targets of ER and CY, we performed a comprehensive search across three public databases: SymMap [38], HERB 2.0 [39], and HIT [40]. Specifically, for the SymMap database, targets were selected based on a statistical significance threshold of *p* < 0.05 to ensure high confidence. All identified targets were then pooled and standardized for further analysis. The intersection of herb- and compound-related targets and CIPN-related targets was identified using a Venn diagram. To understand the interactions among these overlapping targets, a Protein-Protein Interaction (PPI) network was constructed using the STRING database (https://string-db.org/) with a medium confidence score (>0.4). The network was visualized using Cytoscape software (Version 3.10.4). To identify the core targets within the network, the top hub genes were ranked using the Maximal Clique Centrality (MCC) algorithm via the CytoHubba plugin.

### 2.3. Animals

Male C57BL/6J mice (6–8 weeks old) were obtained from Daehan Biolink (DBL, Chungbuk, Republic of Korea). Upon arrival, all animals were housed in standard polycarbonate cages in a temperature- and humidity-controlled environment (23 ± 2 °C; 65 ± 5% relative humidity) under a 12 h light/dark cycle with free access to food and water. To minimize stress-related variability, mice were allowed to acclimate to the housing conditions for one week before any experimental procedures. All behavioral assessments were carried out during the same time period each day to avoid circadian rhythm-related bias. All procedures strictly adhered to the institutional guidelines for animal care and use, as approved by the Institutional Animal Care and Use Committee of Kyung Hee University (IACUC approval numbers: KHUASP(SE)-23-223 [7 August 2024] and KHUASP-24-411 [20 May 2024]). At the end of the experiments, mice were deeply anesthetized and euthanized by inhalation of excessive isoflurane in accordance with ethical standards.

### 2.4. Preparation of Herbal Extracts

Dried rhizomes of CY and fruits of ER were purchased from a commercial supplier (Republic of Korea) and authenticated by morphological inspection and ITS gene analysis. Each herbal material was extracted by the reflux method with 30% ethanol at 80 °C for 6 h. After extraction, the solution was filtered through standard filter paper to remove insoluble residues. The filtrate was concentrated under reduced pressure at 60 °C using a rotary evaporator and subsequently lyophilized with a freeze dryer to obtain a dry powdered extract. For in vivo administration, each extract powder was reconstituted in sterile phosphate-buffered saline (PBS) immediately prior to use. The final suspension was administered to mice orally at a constant volume of 0.1 mL/kg per animal for each designated concentration.

### 2.5. Administration of Paclitaxel, CY, ER, THP, and Rutaecarpine

Paclitaxel (Paclitaxel; Sigma-Aldrich, St. Louis, MO, USA) was first dissolved in a 1:1 mixture of Cremophor EL and absolute ethanol to obtain a stock solution with a concentration of 6 mg/mL. Immediately prior to administration, the stock was further diluted with sterile phosphate-buffered saline (PBS) to a final concentration of 0.2 mg/mL. Mice in the control group received the same volume of vehicle consisting of Cremophor EL–ethanol (1:1) diluted with PBS, without the active compound. Paclitaxel or vehicle was administered by intraperitoneal (i.p.) injection at a dose of 2 mg/kg on experimental D0, D2, D4, and D6 resulting in a total cumulative dose of 8 mg/kg. This dosing schedule was used to reliably induce mechanical and cold allodynia in mice. For herbal treatment, CY and ER extracts were prepared in PBS and administered orally via a feeding sonde (Jungdo-BNP, Seoul, Republic of Korea) at doses of 100 or 300 mg/kg on days 2, 4, 6, and 8 following paclitaxel injection. In addition to single-extract administration, a combination of both extracts was orally co-administered at 100 + 100 mg/kg and 300 + 300 mg/kg to evaluate potential synergistic effects. Based on HPLC quantification, the equivalent alkaloid contents corresponding to the 300 mg/kg extract dose were calculated. Accordingly, rutaecarpine was administered intraperitoneally at 0.23 mg/kg, and THP was administered intraperitoneally at 0.55 mg/kg to evaluate the direct analgesic effects of these purified alkaloids.

### 2.6. Behavioral Test

Cold and mechanical allodynia were evaluated using the acetone drop and von Frey filament test methods, respectively. All behavioral assessments were conducted in a quiet room under controlled temperature and humidity. Prior to testing, the animals were placed on a steel wire mesh platform (behavioral chamber) and acclimated for 30 min to minimize stress-induced variability. For the assessment of cold allodynia, 10 µL of acetone was gently applied to the mid-plantar surface of the hind paw using a pipette. Pain-related behaviors—including paw withdrawals, flinching, and licking—were observed and counted for 30 s following application. The behavioral data were expressed as the average number of responses (“# of Response”) per group.

Mechanical allodynia was assessed using von Frey filaments (Stoelting, Chicago, IL, USA) of varying stiffness (0.02, 0.04, 0.07, 0.16, 0.4, 0.6, 1, 1.4, and 2 g). Filaments were applied perpendicularly to the mid-plantar region of the hind paw with enough force to bend the filament for approximately 2–3 s. The testing sequence followed the up–down method of Steven R. Chaplan and Dixon to determine the 50% paw withdrawal threshold. A positive response (withdrawal or flinch) resulted in the use of a filament with lower force, whereas a negative response led to the application of a higher force filament. The threshold value was calculated according to standard methods. At the end of behavioral testing, animals were deeply anesthetized with isoflurane and euthanized by intracardiac perfusion with cold PBS. Blood was collected from the left ventricle, and the lumbar 4–5 segments of the spinal cord as well as paw tissues were harvested for further molecular analyses (e.g., PCR and Western blotting).

### 2.7. Tissue Collection and Perfusion

For tissue collection, mice designated for immunohistochemistry (IHC), quantitative real-time PCR (qPCR), and high-performance liquid chromatography (HPLC) were deeply anesthetized with inhaled isoflurane until loss of reflexes was observed. Once fully anesthetized, animals underwent transcardial perfusion with cold phosphate-buffered saline (PBS, pH 7.2) until the outflow was visually free of blood. For IHC, brains were carefully dissected following perfusion and immersed in 4% paraformaldehyde (PFA) for fixation. For molecular analyses (qPCR and HPLC), animals were perfused without PFA to avoid fixation of nucleic acids or metabolites. The collected tissues were snap-frozen and stored at −80 °C until further processing.

### 2.8. Quantitative Real-Time Polymerase Chain Reaction (qRT-PCR)

Total ribonucleic acid (RNA) was extracted from the lumbar spinal cord and ipsilateral paw tissues using the AccuPrep RNA extraction kit (Bioneer, Daejeon, Republic of Korea) following the manufacturer’s protocol. RNA concentration and purity were assessed using a NanoDrop spectrophotometer (Thermo Scientific, Waltham, MA, USA), and only samples with OD260/OD280 ratios above 1.8–2.0 were used. Subsequently, 0.5 µg of total RNA from each sample was reverse transcribed into complementary DNA (cDNA) using the Maxime RT PreMix kit (Intron Biotechnology, Seongnam, Republic of Korea). Quantitative real-time PCR (qRT-PCR) was performed using a SensiFAST SYBR Hi-ROX kit (Meridian Bioscience, Cincinnati, OH, USA) on a CFX Real-Time PCR Detection System (Bio-Rad, Hercules, CA, USA). The thermal cycling conditions were as follows: initial denaturation at 95 °C for 5 min, followed by 40 cycles of 95 °C for 20 s, 58 °C for 20 s, and 72 °C for 20 s. For amplification of the *TRPV1* gene, the following primers were used: forward 5′-GGC TGT CTT CAT CAT CCT GCT GCT-3′ and reverse 5′-GTT CTT GCT CTC CTG TGC GAT CTT GT-3′. GAPDH was amplified as an internal reference gene. The relative expression level of *TRPV1* mRNA was calculated using the 2^−ΔΔCt^ method, and values were normalized to GAPDH, with the control group set to 1 [41].

### 2.9. Protein Expression by Western Blot

Protein expression of TRPV1 in spinal cord tissues was analyzed by Western blotting. Tissues were homogenized in radioimmunoprecipitation assay (RIPA) buffer and centrifuged at 13,000 rpm for 10 min at 4 °C. The supernatant was collected, and protein concentrations were determined using a Bradford protein assay kit (Bradford Protein Assay Kit, Bio-Rad, Hercules, CA, USA). Equal amounts of protein (20–30 μg) were separated by sodium dodecyl sulfate–polyacrylamide gel electrophoresis (SDS–PAGE) using 12% gels and transferred to nitrocellulose membranes at 110 V for 100 min. The membranes were blocked with 5% non-fat skim milk in Tris-buffered saline containing 0.1% Tween-20 (TBS-T) for 1 h at room temperature and incubated overnight at 4 °C with primary antibodies: rabbit polyclonal anti-TRPV1 (1:1000, cat. NB100-1617, Novus Biologicals, Littleton, CO, USA), TRPM8 (1:1000, cat. NB1-97311, Novus Biologicals, Littleton, CO, USA) and rabbit polyclonal anti-β-actin (1:1000, cat. PA1-183, Invitrogen, Waltham, MA, USA). After washing three times with TBS-T, the membranes were incubated with horseradish peroxidase (HRP)-conjugated anti-rabbit secondary antibody (1:5000, cat. 31460, Thermo Scientific, Waltham, MA, USA) for 1 h at room temperature. Immunoreactive bands were visualized using an enhanced chemiluminescence detection kit (D-Plus ECL Femto System, Hwaseong, Republic of Korea), and the signal intensity was captured and quantified with Davinch-Chemi software ver. 2.0 (Young-Hwa Science, Daejeon, Republic of Korea). Actin served as the internal loading control for normalization.

### 2.10. Assay Identification and Quantification of THP in CY and Rutaecarpine in ER

The compounds of CY and ER extracts were analyzed using HPLC equipped with a UV detector (Agilent 1260, Santa Clara, CA, USA). The analytical conditions for THP, rutaecarpine, and the herbal extracts are listed in Table 1. A stock solution of THP and rutaecarpine was prepared in methanol. The THP and rutaecarpine standards used in this study were purchased from ChemFace (China). For both standards, five serial dilutions were prepared and analyzed. A total of 100 mg of each extract powder was ultrasonically extracted at 4 °C for 120 min using 1 mL of ethanol. After centrifugation (4 °C, 13,000 rpm, 30 min), the supernatant was filtered through a 0.45 μm syringe filter and used for analysis.

### 2.11. Identification of Potential Targets for THP and Rutaecarpine and Construction of the PPI Network

Potential therapeutic targets for CIPN were collected from the GeneCards database (https://www.genecards.org/) with a relevance score threshold to ensure data quality Stelzer, 2016 [37]. The predicted targets of THP and Rutaecarpine were obtained from databases such as Swiss Target Prediction.

The intersection of compound-related targets and CIPN-related targets was identified using a Venn diagram. To understand the interactions among these overlapping targets, a PPI network was constructed using the STRING database (https://string-db.org/) with a medium confidence score (>0.4) [42]. The network was visualized using Cytoscape software (Version 3.10.4) [43]. To identify the core targets within the network, the top hub genes were ranked using the Maximal Clique Centrality (MCC) algorithm via the CytoHubba plugin.

### 2.12. KEGG Pathway Enrichment Analysis and Molecular Docking Simulation

To elucidate the biological mechanisms and signaling pathways modulated by the candidate compounds, Kyoto Encyclopedia of Genes and Genomes (KEGG) pathway enrichment analysis was performed on the identified common targets. The analysis was conducted using the DAVID functional annotation tool or equivalent bioinformatics resources [44]. Pathways with a *p*-value < 0.05 were considered statistically significant. The top enriched signaling pathways were visualized as bubble plots, with the bubble size representing gene counts and color gradients indicating statistical significance (−log10 FDR). To validate the direct binding affinity between the active compounds and the core therapeutic targets, molecular docking simulations were performed using the CB-DOCK2 server (http://clab.labshare.cn/cb-dock2/ (accessed on 26 November 2025)) [45]. This curvature-based cavity detection method was used for blind docking.

### 2.13. Statistical Analysis

All statistical analyses were carried out using GraphPad Prism (version 9.0; GraphPad Software, San Diego, CA, USA). Data are expressed as mean ± standard deviation (SD) to reflect inter-individual variability. The normality of the data distribution was assessed using the D’Agostino–Pearson omnibus test prior to further analyses. For behavioral experiments, differences between groups were evaluated using two-way analysis of variance (ANOVA) followed by Tukey’s multiple comparison test. Gene and protein expression data were analyzed using one-way ANOVA with Tukey’s post hoc test. Unpaired or paired *t*-tests were applied where appropriate, depending on the experimental design. A *p*-value of less than 0.05 was considered statistically significant for all analyses.

## 3. Results

### 3.1. Shared Targets Selected from Active Components of CY and ER and KEGG Pathway Enrichment Analysis for CIPN

**Figure 1:**
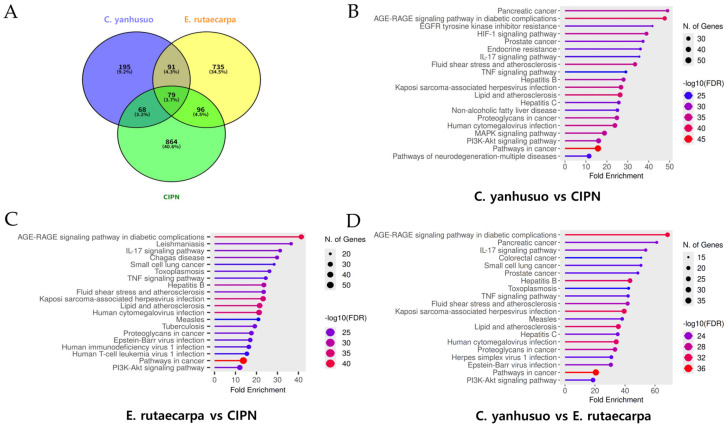
Overlapping targets of CY, ER, and CIPN, and KEGG pathway enrichment analysis. Venn diagram illustrating the overlap among predicted targets of CY, ER, and CIPN-related genes (**A**). A total of 79 common targets were identified at the intersection, suggesting shared molecular mechanisms potentially relevant to chemotherapy-induced peripheral neuropathy. KEGG pathway enrichment analysis of CIPN-related targets associated with CY (**B**). KEGG pathway enrichment analysis of CIPN-related targets associated with ER (**C**). KEGG pathway enrichment analysis of the 79 shared targets between CY and ER (**D**).

We identified putative targets of CY and ER using SymMap (*p* < 0.05), HERB 2.0, and HIT databases, while CIPN-related targets were retrieved from GeneCards. As shown in Figure 1, Venn analysis revealed 79 shared therapeutic targets, suggesting a convergent molecular basis for the analgesic potential of the two herbs. To examine how these targets functionally interact, a PPI network was generated using STRING and visualized in Cytoscape (Figure 1B–D). The dense connectivity within the network indicates that the herbal compounds may act by modulating multiple inflammatory and stress-related proteins simultaneously. KEGG enrichment analysis further highlighted key pathways, including AGE–RAGE, IL-17, TNF, and PI3K–Akt signaling. Enrichment of AGE–RAGE and TNF pathways suggests potential anti-inflammatory and antioxidative actions relevant to CIPN, while PI3K–Akt involvement points to neuroprotective effects that support neuronal survival and repair.

### 3.2. Analgesic Effect of CY and ER on Cold and Mechanical Allodynia Induced by Paclitaxel

**Figure 2:**
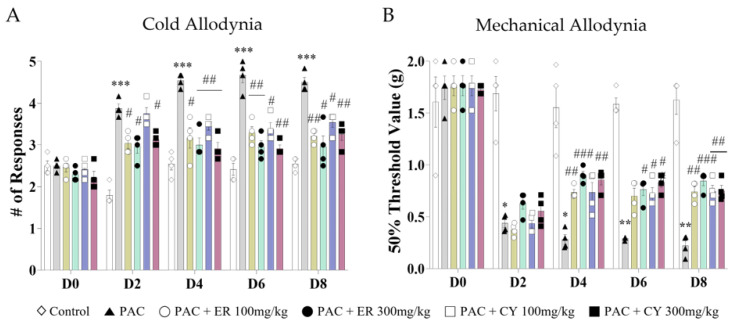
Effect of oral administration of ER and CY on cold and mechanical allodynia (**A**,**B**). Induction of allodynia was injected 4 times as a concentration of 2 mg/kg (an accumulative dose of 8 mg/kg, i.p.), and assessment was performed on D0, D2, D4, D6, D8. ER and CY administration via gavage at concentrations of 100 and 300 mg/kg. PBS was used as a vehicle for extracts and extracts or PBS was orally administered. PAC; paclitaxel, ER; *Evodia rutaecarpa*, CY; *Corydalis yanhusuo*. Data are presented as mean ± SD. *N* = 6 for each group; * *p* < 0.05, ** *p* < 0.01, *** *p* < 0.001 vs. control group and # *p* < 0.05, ## *p* < 0.01, ### *p* < 0.001 vs. paclitaxel group with two-way ANOVA followed by Tukey’s multiple comparison test.

To assess the analgesic effects of CY and ER, each extract was orally administered at 100 and 300 mg/kg in a paclitaxel-induced neuropathic pain model. As shown in Figure 2A, paclitaxel significantly increased cold allodynia compared with controls. Both extracts at 300 mg/kg produced clear analgesic effects, whereas the 100 mg/kg dose showed partial or time-dependent improvements. Although not all comparisons reached statistical significance, both treatments consistently reduced cold hypersensitivity.

For mechanical allodynia (Figure 2B), a weaker but similar trend was observed. Significant analgesic effects were detected only at 300 mg/kg, while the lower dose did not reach significance. Nevertheless, both doses showed a consistent reduction in hypersensitivity. Overall, CY and ER alleviated paclitaxel-induced allodynia in a dose-dependent manner, with more pronounced effects on cold than mechanical sensitivity.

### 3.3. Analgesic Effect of Combined CY and ER Administration on Cold and Mechanical Allodynia Induced by Paclitaxel

**Figure 3:**
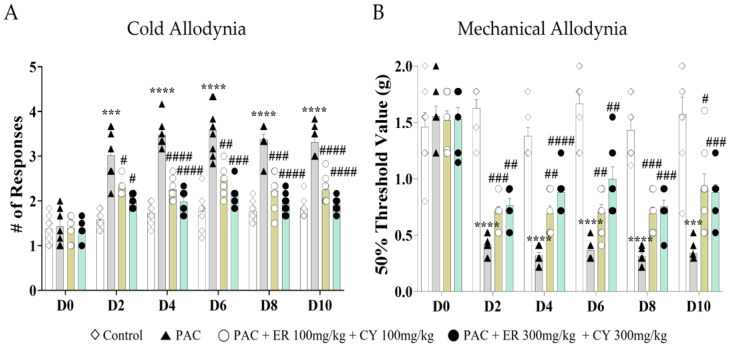
Effect of combined oral administration of ER and CY on cold and mechanical allodynia (**A**,**B**). Induction of allodynia was injected 4 times as a concentration of 2 mg/kg (an accumulative dose of 8 mg/kg, i.p.), and assessment was performed on D0, D2, D4, D6, D8 and D10. ER and CY were co-administered via gavage, each at doses of 100 or 300 mg/kg. PBS was used as a vehicle for extracts and extracts or PBS was orally administered. PAC; paclitaxel, ER; *Evodia rutaecarpa*, CY; *Corydalis yanhusuo*. Data are presented as mean ± SD. *N* = 6 for each group; *** *p* < 0.001, **** *p* < 0.0001 vs. control group and # *p* < 0.05, ## *p* < 0.01, ### *p* < 0.001, #### *p* < 0.0001 vs. paclitaxel group with two-way ANOVA followed by Tukey’s multiple comparison test.

Paclitaxel administration significantly increased cold and mechanical allodynia compared with the control group. As shown in Figure 3A, oral administration of CY or ER at 100 and 300 mg/kg significantly reduced cold hypersensitivity, with greater effects observed at 300 mg/kg. Combination treatment further enhanced the reduction in cold allodynia, and the 300 + 300 mg/kg group showed the greatest attenuation across all time points. Similarly, mechanical allodynia was significantly attenuated by CY or ER treatment in a dose-dependent manner (Figure 3B). Administration of 300 mg/kg of each extract produced stronger effects than 100 mg/kg, and both combination groups (100 + 100 mg/kg and 300 + 300 mg/kg) significantly reduced mechanical hypersensitivity compared with single administration. Overall, combination treatment produced greater anti-allodynic effects than individual extracts, with more pronounced effects observed in cold allodynia.

### 3.4. Effects of ER and CY on Paclitaxel-Induced TRPV1, TRPM8, and TRPA1 Expression and Cell Viability

**Figure 4:**
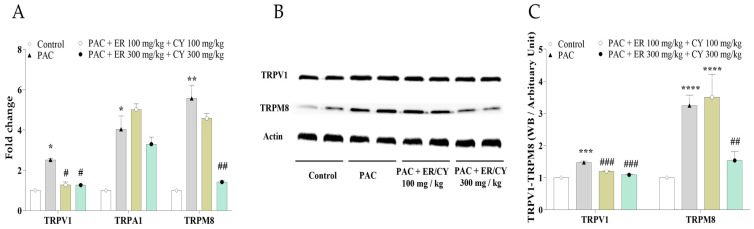
Quantitative RT-PCR analysis of *TRPV1*, *TRPM8*, and *TRPA1* mRNA expression in the spinal cord (**A**) following paclitaxel administration and ER + CY treatment. Paclitaxel (2 mg/kg, i.p., four injections; cumulative dose 8 mg/kg) was administered on D0, D2, D4, and D6. ER and CY were orally administered via gavage at 100 or 300 mg/kg, either alone or in combination. Spinal cord tissues were collected on D10 for mRNA analysis. PBS was used as the vehicle. Western blot analysis of TRPV1 and TRPM8 protein expression (**B**,**C**) in the spinal cord collected on D10. PAC; paclitaxel, ER; *Evodia rutaecarpa*, CY; *Corydalis yanhusuo*. * *p* < 0.05, ** *p* < 0.001, *** *p* < 0.001, **** *p* < 0.0001 vs. control group and # *p* < 0.05, ## *p* < 0.01, ### *p* < 0.001 vs. paclitaxel group with two-way ANOVA followed by Tukey’s multiple comparison test.

Paclitaxel administration significantly increased *TRPV1*, *TRPM8*, and *TRPA1* mRNA expression, confirming the establishment of a neuropathic pain-related molecular profile. Co-administration of ER and CY (100 or 300 mg/kg) effectively reversed this effect by significantly reducing *TRPV1* and *TRPM8* mRNA levels, whereas *TRPA1* showed only a mild, non-significant decrease, indicating a limited regulatory response for this gene (Figure 4A). Western blot analysis further supported these findings, showing that paclitaxel-induced increases in TRPV1 and TRPM8 protein expression were markedly suppressed by ER + CY treatment, demonstrating normalization of these nociceptive ion channels at the protein level. TRPA1 protein expression was not evaluated, and its regulation was assessed only at the transcriptional level (Figure 4B,C).

### 3.5. Identification and Quantification of Rutaecarpine in ER and Tetrahydropalmatine in CY

**Figure 5:**
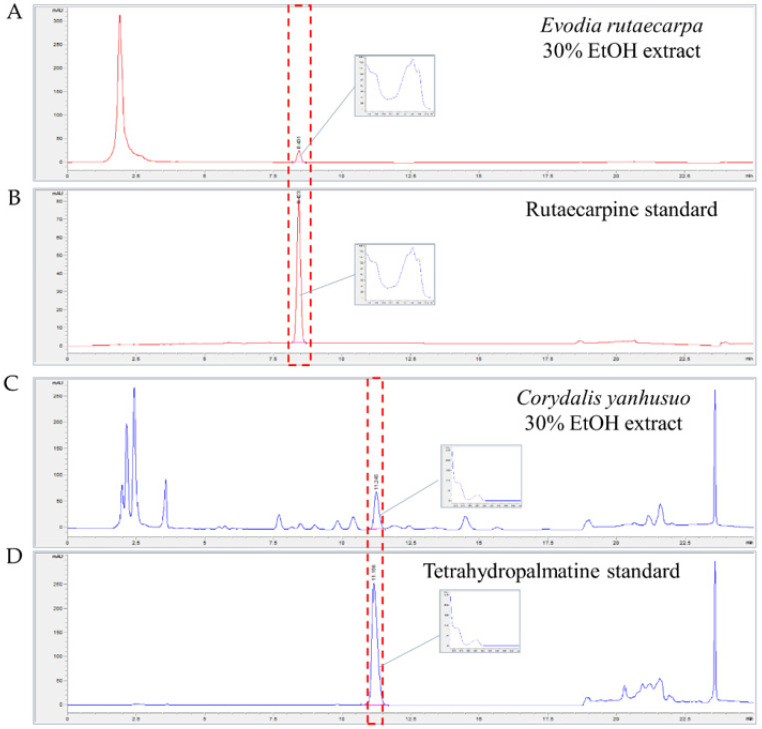
Quantification and identification of rutaecarpine and THP by high performance liquid chromatography (HPLC). HPLC chromatograms of ER extract (**A**), rutaecarpine standard (**B**), CY extract (**C**), and THP standard (**D**). The red dashed boxes mark the characteristic peaks that appear in both the plant extracts and their respective reference standards, respectively. Retention time and absorbance unit are shown on the X-axis and Y-axis, respectively.

HPLC analysis was performed to identify and quantify THP in *Corydalis yanhusuo* and rutaecarpine in *Evodia rutaecarpa* extracts. The CY 30% EtOH extract exhibited a chromatographic peak matching the retention time and UV spectrum of the THP standard (Figure 5A–D), with a quantified content of 0.183%. In the ER extract, a peak corresponding to rutaecarpine was detected at the same retention time as the reference standard, and its content was determined to be 0.0775%. These results confirm the presence of THP in CY and rutaecarpine in ER.

### 3.6. Analgesic Effects of Rutaecarpine, THP, and Their Combination on Paclitaxel-Induced Cold and Mechanical Allodynia in Mice

**Figure 6:**
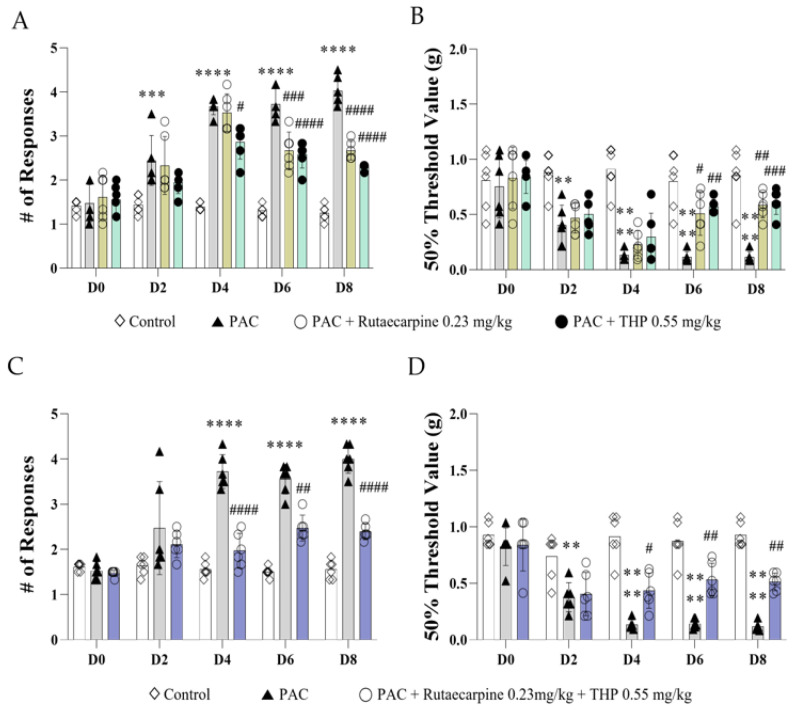
Analgesic effects of rutaecarpine and THP on paclitaxel-induced cold and mechanical allodynia in mice. Cold (**A**,**B**) and mechanical allodynia (**C**,**D**) were evaluated before and after intraperitoneal administration of rutaecarpine, THP, or their combination. Mice received vehicle or paclitaxel (PAC, 2 mg/kg, i.p., four injections; cumulative dose 8 mg/kg), followed by gavage of rutaecarpine, THP, or the rutaecarpine–THP combination at doses of 0.23 or 0.55 mg/kg. PAC; paclitaxel, THP; tetrahydropalmatine. Data are presented as mean ± SD. *N* = 6 for each group; ** *p* < 0.01, *** *p* < 0.001, **** *p* < 0.0001 vs. control group and # *p* < 0.05, ## *p* < 0.01, ### *p* < 0.001, #### *p* < 0.0001 vs. paclitaxel group with two-way ANOVA followed by Tukey’s multiple comparison test.

Paclitaxel administration significantly increased cold and mechanical allodynia compared with the control group. As shown in Figure 6A,B, single intraperitoneal administration of rutaecarpine (0.23 mg/kg) or THP (0.55 mg/kg) significantly attenuated both types of allodynia. Moreover, co-administration of rutaecarpine and THP produced greater analgesic effects than either compound alone (Figure 6C,D). These results indicate that both alkaloids exert significant anti-allodynic effects individually, while their combined administration leads to enhanced analgesic efficacy.

### 3.7. Integrated Analysis of Overlapping Targets and KEGG Pathway Enrichment for THP and Rutaecarpine in CIPN

**Figure 7:**
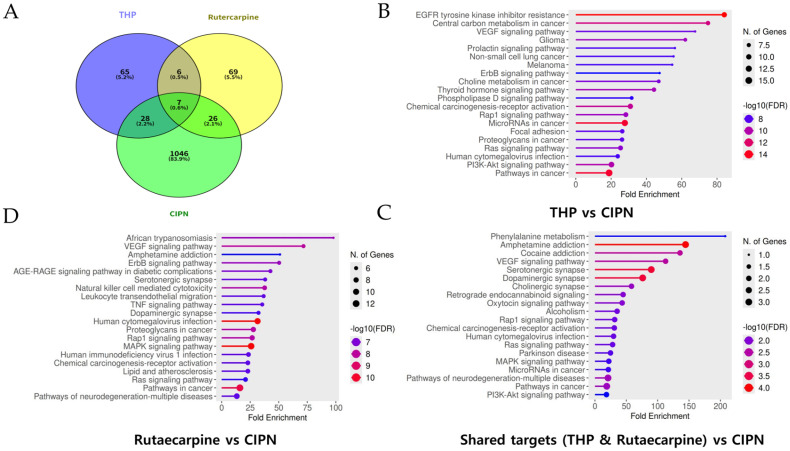
Integrated analysis of overlapping targets and KEGG pathway enrichment for THP and rutaecarpine in CIPN. Venn diagram illustrating the overlapping predicted targets of THP, rutaecarpine, and CIPN (**A**). A total of 7 shared targets were identified at the intersection of all three datasets. KEGG pathway enrichment analysis of potential therapeutic targets against CIPN. (**B**) Top enriched KEGG signaling pathways associated with THP targets related to CIPN (**C**). Top enriched KEGG signaling pathways associated with rutaecarpine targets related to CIPN (**D**). Top enriched KEGG signaling pathways derived from the shared targets between THP and rutaecarpine. In all bubble plots, the x-axis represents the fold enrichment ratio, and the y-axis lists the corresponding pathway categories. The bubble size indicates the number of genes involved (Gene Count), while the color gradient denotes statistical significance expressed as −log10(FDR).

Although TRPV1 and TRPM8 were not identified as direct targets in the network pharmacology analysis (Figure 7A), KEGG enrichment revealed multiple pathways known to regulate TRP channel sensitization and nociceptive signaling. As shown in Figure 7B–D, significantly enriched pathways included MAPK, PI3K–Akt, Rap1/Ras signaling, and several synaptic pathways. These pathways are established upstream regulators of TRPV1 and TRPM8 and are implicated in chemotherapy-induced peripheral neuropathy. In addition, addiction- and neurodegeneration-related pathways, which involve calcium influx and neuronal hyperexcitability, were commonly enriched across THP, rutaecarpine, and shared target sets. Collectively, these findings suggest that THP and rutaecarpine may indirectly modulate TRP channel-related nociceptive processes despite the absence of direct TRPV1/TRPM8 mapping in the target overlap.

### 3.8. Docking Analysis of THP and Rutaecarpine on TRP Channels

**Figure 8:**
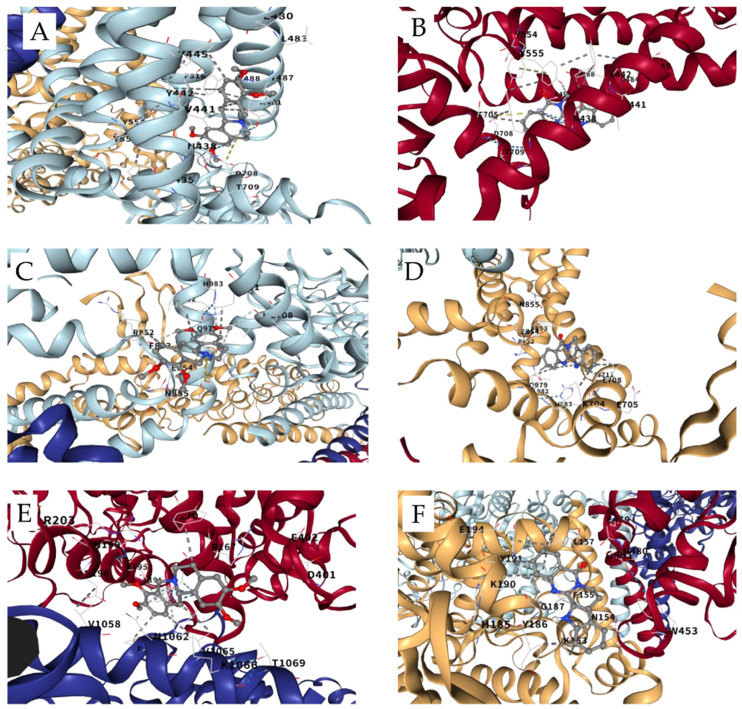
Molecular docking simulation of THP and rutaecarpine with TRP ion channels. Structure-based blind docking was performed using the CB-Dock2 server to predict binding modes and affinities. Docking poses of THP and rutaecarpine with TRPV1 (PDB:8GFA). Both ligands showed strong binding affinities (−9.4 and −9.7 kcal / mol, respectively) (**A**,**B**). Interaction with TRPA1 (PDB:7OR1). Rutaecarpine exhibited the highest binding affinity (−9.8 kcal/mol) at cavity 1 (C1), while THP showed moderate affinity (−7.5 kcal/mol) at cavity 2 (C2) (**C**,**D**). Docking results for TRPM8 (PDB:8BDC). Rutaecarpine maintained strong binding (−9.1 kcal/mol), whereas THP showed relatively lower affinity (−7.9 kcal / mol) (**E**,**F**). Abbreviations: THP, l-tetrahydropalmatine; C1/C2, binding cavity rank.

Molecular docking analysis using CB-Dock2 (Table 2) showed that THP and rutaecarpine exhibit distinct predicted binding affinities toward TRPV1, TRPA1, and TRPM8. Both compounds displayed strong interactions with TRPV1, with rutaecarpine showing the highest affinity (Vina score: −9.7 kcal/mol), followed by THP (−9.4 kcal/mol). The predicted binding poses (Figure 8A,B) indicated stabilization by key residues within the TRPV1 binding pocket, including F543, Y554, and Q560. For TRPA1, rutaecarpine exhibited strong predicted binding (−9.8 kcal/mol), whereas THP showed moderate affinity (−7.5 kcal/mol), consistent with differences in docking orientation (Figure 8C,D). Docking to TRPM8 revealed moderate affinity for THP (−7.9 kcal/mol) and strong affinity for rutaecarpine (−9.1 kcal/mol) (Figure 8E,F). Overall, these results indicate that rutaecarpine consistently shows stronger predicted binding affinities than THP across TRP channels, particularly for TRPV1 and TRPA1, while THP preferentially targets TRPV1.

### 3.9. In Silico ADMET Analysis of THP and Rutaecarpine

In silico ADMET analysis indicated favorable pharmacokinetic properties for both THP and rutaecarpine (Table 3). Predicted human intestinal absorption was high for both compounds (93.10% for THP and 97.29% for rutaecarpine), supporting good oral bioavailability. Rutaecarpine showed high Caco-2 permeability (1.26), whereas THP exhibited moderate permeability (0.68). Both compounds were predicted to cross the blood–brain barrier (logBB: 0.17 for THP; 0.67 for rutaecarpine) and demonstrated CNS permeability within the accepted range (logPS > −2.0). Both THP and rutaecarpine were predicted CYP3A4 substrates, indicating hepatic metabolism. Toxicity predictions revealed no AMES mutagenicity for THP, while rutaecarpine showed a positive AMES signal; neither compound was predicted to inhibit hERG I channels. Both compounds showed potential hepatotoxicity, suggesting safety considerations at higher exposures. Overall, these ADMET profiles support adequate oral absorption and CNS penetration, while highlighting the need for further safety evaluation.

## 4. Discussion

This study employed a network-pharmacology approach to explore how CY and ER may alleviate CIPN. The analysis indicated a multi-target mechanism involving modulation of inflammatory signaling, oxidative stress responses, and neuronal survival pathways. Enrichment of inflammatory and oxidative stress-related pathways suggests that these herbs may attenuate peripheral nerve sensitization and protect against chemotherapy-induced neuronal injury, while neuroprotective pathways imply potential support for neural recovery beyond symptomatic relief.

Paclitaxel-induced peripheral neuropathy is a major clinical challenge, frequently causing cold and mechanical allodynia that impairs quality of life and limits chemotherapy continuation [46,47,48]. Consistent with previous reports [46,47], our model reliably reproduced these symptoms. Oral administration of CY and ER extracts significantly alleviated paclitaxel-induced hypersensitivity in a dose-dependent manner, with the 300 mg/kg dose showing the strongest antinociceptive effects [49,50,51,52], while lower doses produced partial but consistent improvements, particularly in cold allodynia. These findings support the traditional use of CY and ER for pain relief and extend their relevance to CIPN.

Importantly, co-administration of CY and ER produced greater analgesic effects than either extract alone. This enhancement is consistent with the traditional concept of herb–herb compatibility (e.g., Jin Ling Zi San, Yuanhu-Zhitong) and is increasingly recognized in pharmacological research as additive and/or potentially synergistic interactions [50]. Combination treatment at both 100 + 100 mg/kg and 300 + 300 mg/kg more effectively reduced cold and mechanical allodynia than single administration, suggesting complementary actions of bioactive compounds in CY and ER. The pronounced suppression of cold allodynia is consistent with previous reports implicating TRP-channel dysregulation, including TRPV1, in paclitaxel-induced hypersensitivity [53,54,55,56].

To assess the contribution of major alkaloids, we examined tetrahydropalmatine (THP) from CY and rutaecarpine from ER. Both compounds produced dose-dependent antinociceptive effects, and their combined administration showed the strongest inhibition of allodynia, mirroring the effects observed with combined herbal extracts. These findings suggest that THP and rutaecarpine may represent major contributors to the enhanced efficacy of the combined treatment. Previous studies have shown that THP exerts anti-inflammatory and neuroprotective effects via TLR4/NF-κB and PI3K/AKT signaling [52], while rutaecarpine displays anti-inflammatory and analgesic actions involving TRPV1 modulation and PLC inhibition.

At the molecular level, paclitaxel markedly increased spinal TRPV1 and TRPM8 expression, whereas co-administration of CY and ER attenuated these changes. This modulation of TRP channel-related signaling is consistent with prior reports linking TRP-channel dysregulation to paclitaxel-evoked allodynia and supports its association with the observed antinociceptive effects.

Collectively, our findings are consistent with an association between TRPV1 signaling and the anti-allodynic effects of CY, ER, THP, and rutaecarpine. Paclitaxel has been reported to upregulate TRPV1 expression in spinal neurons, contributing to increased neuronal excitability and hypersensitivity [56]. In addition, glial activation can further sensitize TRPV1-mediated nociceptive signaling, thereby amplifying cold and mechanical stimuli [54]. Consistent with these reports, the behavioral outcomes in our study showed attenuation of hypersensitivity patterns associated with TRPV1-related changes. Although further molecular and functional validation (e.g., electrophysiology or protein-level analyses) is warranted, these findings are in line with previous studies demonstrating relief of paclitaxel-induced neuropathy through TRPV1-targeted mechanisms [57].

Collectively, these findings suggest that CY, ER, and their alkaloids THP and rutaecarpine exhibit therapeutic potential for the management of chemotherapy-induced neuropathic pain. Combination treatment consistently produced greater analgesic efficacy than single administration, indicating that multi-component herbal approaches may offer advantages over isolated compounds. Given the limitations of current neuropathic pain therapies, including dose-limiting toxicity and insufficient efficacy [58,59,60], natural product-based interventions represent a promising complementary strategy. Future studies should further investigate pharmacokinetic interactions, potential additive or synergistic mechanisms involving TRPV1-related pathways, and long-term safety to assess translational feasibility. Nonetheless, the present results provide robust preclinical evidence supporting CY–ER combination formulations as potential natural analgesics for paclitaxel-induced neuropathic pain. In addition to the major alkaloids quantified in this study, several minor peaks were detected in the HPLC chromatograms. Although these components were not structurally identified here, they may also contribute to the overall analgesic activity of the extracts. Future studies employing LC–MS/MS profiling and compound isolation will be needed to characterize these constituents and evaluate their pharmacological relevance.

Network pharmacology and KEGG pathway analyses indicated that THP and rutaecarpine are associated with multiple signaling pathways, including MAPK, PI3K–Akt, Ras/Rap1, and neurotransmitter synapse pathways, which are known upstream regulators of TRPV1 and TRPM8 sensitization. Although TRPV1 and TRPM8 were not directly identified as overlapping targets, enrichment of these regulatory pathways suggests that they may act as functionally relevant downstream mediators in CIPN. Consistent with this interpretation, molecular docking predicted favorable interactions of both compounds with TRPV1, and of rutaecarpine with TRPM8, supporting their potential involvement in TRP channel-related nociceptive modulation. It should be noted that the network pharmacology and molecular docking analyses in this study are intended as hypothesis-generating tools. While these in silico approaches provide supportive evidence for potential target–compound interactions, they do not constitute direct experimental validation of binding or functional modulation.

ADMET predictions further indicated favorable absorption and CNS permeability for both compounds, supporting their ability to access nociceptive pathways. While potential hepatotoxicity and CYP3A4 metabolism were predicted, these findings warrant further evaluation rather than precluding mechanistic interpretation. From a translational perspective, the predicted hepatotoxicity and AMES positivity observed for rutaecarpine highlight important safety considerations. These in silico predictions do not necessarily indicate clinical toxicity but underscore the need for careful dose optimization, metabolic profiling, and long-term safety evaluation in future studies. Notably, the use of whole herbal extracts may mitigate potential toxicity through lower effective concentrations and buffering interactions among multiple constituents, whereas isolated alkaloids may require more stringent toxicological assessment. Therefore, comprehensive pharmacokinetic and toxicity studies will be essential before clinical translation of these compounds. Overall, the integrated network, docking, and ADMET analyses support the relevance of TRPV1- and TRPM8-associated signaling as plausible downstream contributors to THP- and rutaecarpine-mediated analgesic effects in CIPN. In considering therapeutic development, both whole plant extracts and isolated alkaloids present distinct advantages. Whole extracts may exert synergistic or buffering effects among multiple phytochemicals, whereas purified alkaloids provide greater mechanistic specificity and controllable pharmacokinetics. Future comparative studies evaluating efficacy, toxicity, and formulation feasibility will be essential to determine the most suitable approach for clinical translation.

## 5. Conclusions

In summary, this study demonstrates that CY and ER, together with their major alkaloids THP and rutaecarpine, effectively alleviate paclitaxel-induced cold and mechanical allodynia in mice. Both extracts produced clear dose-dependent analgesic effects, and their combination resulted in even greater efficacy than single administration, indicating additive or potentially synergistic interactions. Similarly, THP and rutaecarpine significantly reduced paclitaxel-evoked hypersensitivity, with the combined treatment yielding the most robust anti-allodynic response. HPLC analysis verified the presence of these bioactive components in each extract, supporting their direct contribution to the observed pharmacological actions.

Overall, the analgesic effects of CY, ER, and their representative alkaloids highlight their potential as natural therapeutic options for chemotherapy-induced neuropathic pain. These findings provide foundational evidence that multi-component herbal approaches may offer enhanced efficacy with fewer side effects, and they warrant further investigation into TRPV1/TRPM8-related mechanisms and clinical translation. In particular, normalization of TRPV1 and TRPM8 expression and activity may represent key mechanisms underlying their anti-allodynic effects in CIPN.

## Figures and Tables

**Table 1:** Analytical conditions of HPLC for the tetrahydropalmatine and rutaecarpine analysis.

Conditions						
Treatment			Tetrahydropalmatine			Rutaecarpine
Column			Ymc-Triart C18			Ymc-Triart C18
Flow rate			1.0 mL/min			1.0 mL/min
Injection volume			10 μL			10 μL
UV detection			254 nm			240 nm
Run time			25 min			25 min
Tetrahydropalmatine			Rutaecarpine			Flow
Time (min)	Aceto -nitrile	0.1% Phosphoric acid	Time (min)	Aceto -nitrile	Water	mL/min
0	75	25	0	65	35	1.0
15	70	30	15	65	35	1.0
17	100	0	17	100	0	1.0
20	100	0	19	100	0	1.0
21	75	25	21	65	35	1.0
25	75	25	25	65	35	1.0

**Table 2:** Molecular docking results of THP and Rutaecarpine with TRP channels obtained from CB-Dock2.

Target Receptor	PDB ID	Ligand	Binding Cavity	Vina Score (kcal/mol)	Affinity
TRPV1	8GFA	THP	C2	−9.4	Strong
		Rutaecarpine	C1	−9.7	Very Strong
TRPM8	8BDC	THP	C2	−7.9	Moderate
		Rutaecarpine	C1	−9.1	Strong

**Table 3:** Predicted ADMET properties of THP and Rutaecarpine.

Category	Model Name	THP	Rutaecarpine	Threshold/Interpretation
Absorption	Intestinal absorption (human)	93.10%	97.29%	High absorption (>30%)
	Caco-2 permeability	0.68	1.26	High permeability (>0.90)
Distribution	BBB permeability (logBB)	0.17	0.67	Crosses BBB (>−1)
	CNS permeability (logPS)	−1.83	−1.80	Penetrates CNS (>−2.0)
Metabolism	CYP3A4 substrate	Yes	Yes	Metabolized by CYP3A4
Toxicity	AMES toxicity	No	Yes	THP is non-mutagenic
	hERG I inhibitor	No	No	Low cardiotoxicity risk
	Hepatotoxicity	Yes	Yes	Liver toxicity risk

## Data Availability

The data presented in this study are available on request from the corresponding author.
